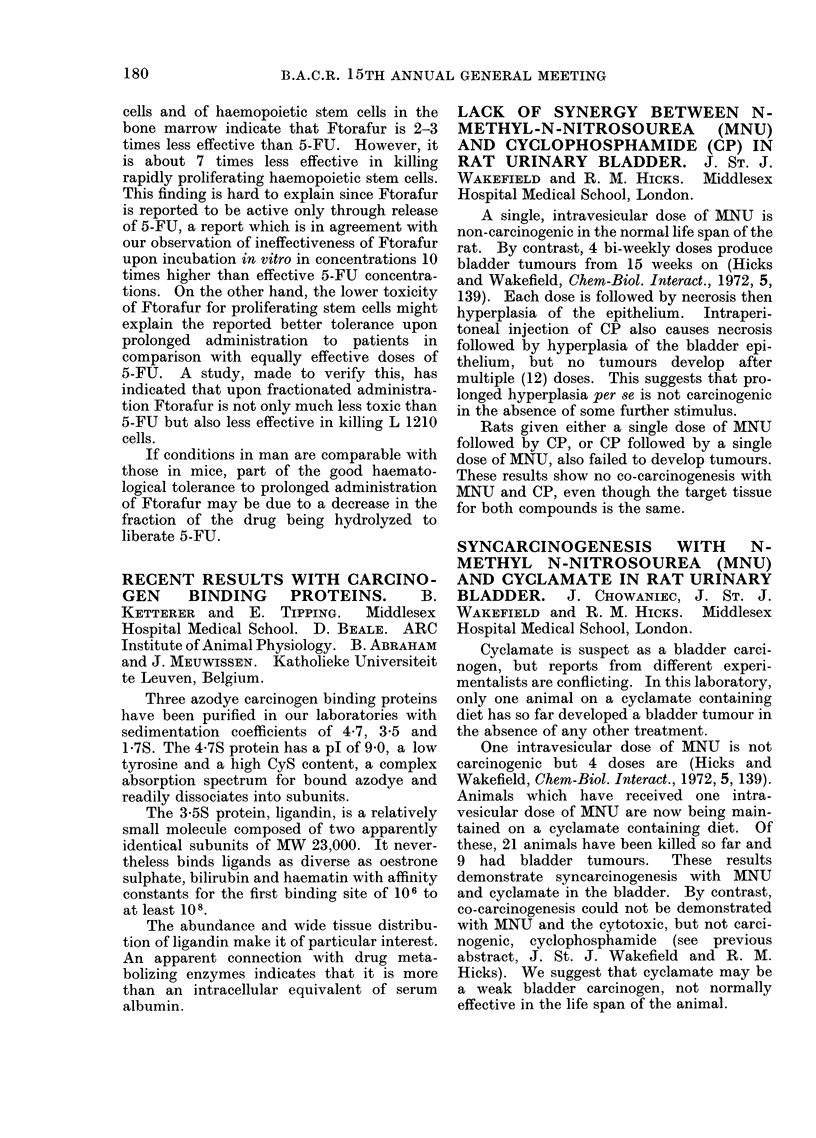# Proceedings: Lack of synergy between n-methyl-N-nitrosourea (MNU) and cyclophosphamide (CP) in rat urinary bladder.

**DOI:** 10.1038/bjc.1974.156

**Published:** 1974-08

**Authors:** J. S. Wakefield, R. M. Hicks


**LACK OF SYNERGY BETWEEN N-METHYL-N-NITROSOUREA (MNU) AND CYCLOPHOSPHAMIDE (CP) IN RAT URINARY BLADDER.** J. St. J. Wakefield and R. M. Hicks. Middlesex Hospital Medical School, London.

A single, intravesicular dose of MNU is non-carcinogenic in the normal life span of the rat. By contrast, 4 bi-weekly doses produce bladder tumours from 15 weeks on (Hicks and Wakefield, *Chem-Biol. Interact.*, 1972, **5**, 139). Each dose is followed by necrosis then hyperplasia of the epithelium. Intraperitoneal injection of CP also causes necrosis followed by hyperplasia of the bladder epithelium, but no tumours develop after multiple (12) doses. This suggests that prolonged hyperplasia *per se* is not carcinogenic in the absence of some further stimulus.

Rats given either a single dose of MNU followed by CP, or CP followed by a single dose of MNU, also failed to develop tumours. These results show no co-carcinogenesis with MNU and CP, even though the target tissue for both compounds is the same.